# Antiepileptic Drugs Impair Shortening of Isolated Cardiomyocytes

**DOI:** 10.3389/fneur.2017.00133

**Published:** 2017-04-03

**Authors:** Johanna Hulbert, Christian E. Elger, Rainer Meyer, Rainer Surges

**Affiliations:** ^1^Institute of Physiology II, University Hospital Bonn, Bonn, Germany; ^2^Department of Epileptology, University Hospital Bonn, Bonn, Germany

**Keywords:** antiepileptic drugs, cardiac block, cardiomyocytes, contraction, epilepsy, side effects, sudden unexpected death in epilepsy

## Abstract

**Background:**

Most antiepileptic drugs (AEDs) inhibit seizure generation by acting on voltage-dependent ion channels. Voltage-dependent sodium and calcium channels are commonly expressed in brain and heart, suggesting that AEDs may have considerable cardiodepressive effects, thereby facilitating sudden cardiac death as a potential cause of sudden unexpected death in epilepsy. Here, we investigated the effects of carbamazepine (CBZ), lamotrigine (LTG), and levetiracetam (LEV) alone and in combination on the shortening properties of isolated ventricular cardiomyocytes of wild-type mice.

**Methods:**

Properties of murine cardiomyocytes were determined by recording the sarcomere shortening with a video imaging system before, during, and after administration of AEDs in different concentrations and combinations. We assessed (i) the number of successful shortenings during continuous electrical stimulation (electromechanical coupling) and (ii) the shortening amplitude as well as other shortening-related properties upon repetitive electrical stimulation at 4 Hz. Data are given as mean ± SEM.

**Results:**

At 100 μM, CBZ (10 cells), LTG (11 cells), and LEV (11 cells) alone had no effect on the electromechanical coupling but reversibly reduced shortening amplitudes by 15 ± 4, 24 ± 3, and 11 ± 3%, respectively. Increasing the LTG concentration to 250 (21 cells) and 500 μM (4 cells) reversibly inhibited the electromechanical coupling in 62 and 100% of the experiments. Importantly, simultaneous application of CBZ, LTG, and LEV at 100 μM also impaired the electromechanical coupling in 8 of 19 cardiomyocytes (42%) and reduced the shortening amplitude by 21 ± 4%.

**Conclusion:**

Our data show that AEDs reversibly impair cardiac excitation and contraction. Importantly, the blocking effect on electromechanical coupling appears to be additive when different AEDs are simultaneously applied. The translational value of these experimental findings into clinical practice is limited. Our results, however, suggest that rationale AED therapy may be important with respect to cardiac side effects and potential facilitation of serious cardiac dysfunction especially when AEDs are used in combination or at very high doses.

## Introduction

In people with epilepsy, the risk of dying prematurely is increased 2.5-fold as compared to the general population (1), about one fifth of these cases are due to sudden unexpected death in epilepsy (SUDEP) (2). The MORTEMUS study has shown that the fatal event is probably due to a severe suppression of cardiorespiratory function shortly after a generalized tonic–clonic seizure in the majority of SUDEP cases (3). Given the large spectrum of epilepsy-related cardiorespiratory alterations, however, SUDEP is likely to be a heterogeneous phenomenon and to have multiple causes (4). For instance, SUDEP may also occur in the absence of epileptic seizures with a similar pattern as described in the MORTEMUS study (5). It is also plausible that, in some cases, SUDEP is due to fatal cardiac arrhythmias such as bradyarrhythmias or ventricular tachyarrhythmias that are not directly related to epileptic seizures. This assumption is supported by several facts: (i) people with epilepsy frequently display abnormal cardiac and autonomic features in the absence of epileptic seizures (6), (ii) epilepsy is a risk factor for sudden cardiac death in the general population (7), (iii) sudden cardiac death occurred in people with epilepsy without evidence of precedent epileptic seizures (8), and (iv) about 10% of witnessed SUDEP cases were reported without apparent seizure activity (9).

The apparent propensity to cardiac mortality and sudden cardiac death in people with epilepsy may have genetic or acquired, epilepsy-related causes. Furthermore, antiepileptic drugs (AEDs) appear to be an independent risk factor for sudden cardiac death, especially in those patients taking AEDs that target voltage-gated sodium channels (VGSC) (10, 11). Numerous experimental studies have revealed that many AED predominantly act on excitatory VGSC and voltage-gated calcium channels (VGCC) or interact with ligand-gated chloride- and mixed-cation channels. A single AED, however, can bind to multiple targets (12, 13). For instance, the drugs that are usually considered as “sodium channel blockers” such as carbamazepine (CBZ), lamotrigine (LTG), and phenytoin also inhibit high-threshold VGCC, whereas levetiracetam (LEV) appears to mediate its antiepileptic effects *via* binding to the presynaptic synaptic vesicle protein 2A but also acts on high-threshold VGCC (12, 13).

Voltage-gated sodium channels and VGCC are expressed both in the brain and heart. The different subtypes of these channels, however, display predominant expression patterns, e.g., within cerebral or cardiac tissue. For instance, the VGSC subtypes NaV1.1, 1.2, 1.3, and 1.6 are primarily responsible for neuronal depolarization in the brain, whereas NaV1.4 and 1.5 are predominantly found in skeletal and cardiac muscle (14). The VGCC subtypes CaV1.2 and 1.3 as well as CaV3.1 and 3.2 are expressed in neurons and cardiomyocytes (15).

In our study, we wanted to examine the effects on cardiac tissue of three selected AEDs alone and in combination. We have chosen CBZ as it is considered the prototype of sodium channel blockers used in the treatment of people with epilepsy. LEV and LTG were selected, because they are nowadays the most widely used AEDs in the developed countries (16). Furthermore, CBZ and LTG were discussed as potential risk factors for SUDEP in previous studies (17). As a single AED can modulate both VGSC and VGCC, we have not investigated effects on a selected molecular target (e.g., by performing patch-clamp recordings to measure specific ion currents) but have recorded the shortening of isolated cardiomyocytes upon electrical stimulation. This experimental approach does not allow the attribution of the observed effects to a specific molecular target but represents the sum of all pathways that are involved in the excitation and shortening of cardiomyocytes. We have assessed electromechanical coupling and the shortening properties upon application of CBZ, LTG, and LEV alone and in combination.

## Materials and Methods

### Animals

C57BL/6 mice of male sex (Charles River/Breeding in the Department of Anatomy at the University of Bonn) were housed at 22°C with 12-h light–dark cycle and fed with water and food *ad libitum*. Twelve- to sixteen-week-old mice were used for the experiments. Animals were anesthetized using isoflurane in room air and euthanized by cervical dislocation.

### Murine Cardiomyocyte Isolation

Myocytes were isolated as previously described (18). The hearts were removed and transferred to a cold cardioplegic EGTA-Tyrode solution (NaCl 135 mM, KCl 4 mM, MgCl_2_ 1 mM, HEPES 2 mM, and EGTA 0.1%, pH 7.4). The removed hearts were rapidly attached to a modified Langendorff apparatus and perfused with oxygenated and heated solutions (35–36°C). Hearts were firstly perfused with EGTA-Tyrode, then with a high-potassium solution (NaCl 4 mM, KCl 10 mM, MgCl_2_ 1 mM, CaCl_2_ 0.025 mM, K-glutamate 130 mM, HEPES 4 mM, glucose monohydrate 9 mM), followed by high-potassium solution supplemented with trypsin (150 U/ml Type I, Sigma-Aldrich) and finally by high-potassium solution supplemented with collagenase (16 U/ml—caseinase activity—Sigma blend Type L, Sigma-Aldrich). Afterward, the ventricles were cutoff the Langendorff apparatus, finely minced and completely dismantled by stirring with a glass bar inside experimental Tyrode [NaCl 135 mM, KCl 4 mM, MgCl_2_ 1 mM, CaCl_2_ 1.8 mM, HEPES 2 mM, glucose monohydrate 10.1 mM, bovine serum albumin 0.1%, and trypsin inhibitor (Type II-S Soybean 1.67 mg/100 ml, pH 7.4)]. The solution was filtered through a 125-μm nylon mesh and centrifuged slowly for about 10 s. The cells were resuspended in fresh solution, allowed to settle for 7 min (heating cabinet 36°C) and resuspended again. Isolated cells were kept in oxygenated experimental Tyrode solution at room temperature for up to 6 h.

### Sarcomere Shortening

**Figure 1:**
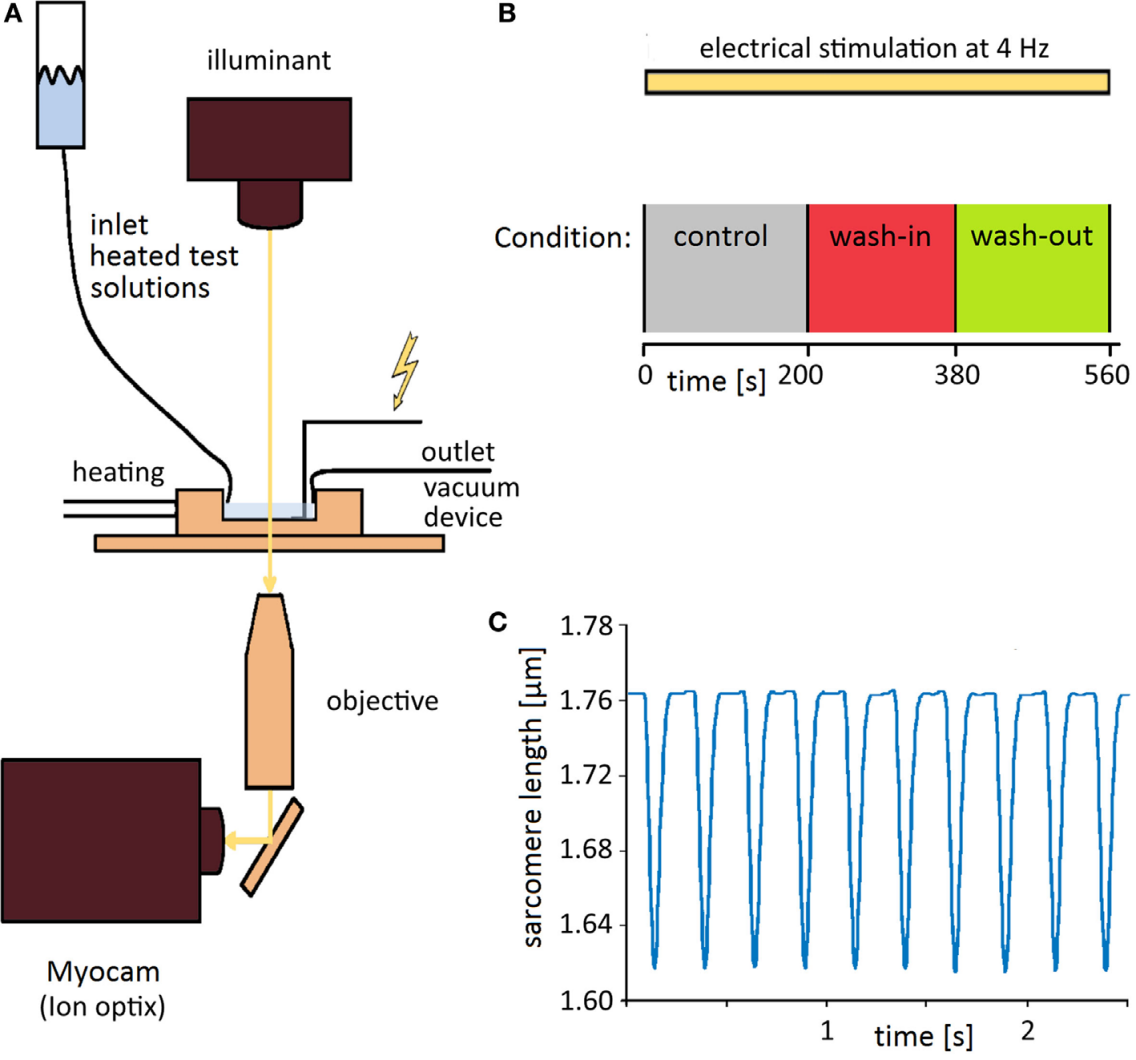
**Setup and time course of experiments**. **(A)** Experimental setup allowing optical measurement of sarcomere lengthening (see Materials and Methods for details). **(B)** A scheme of the time course of experiments. Shortenings were elicited by regular stimulations at 4 Hz before, during, and after wash-out of the test solutions. **(C)** Example of recording traces. The sarcomere length shortens upon extracellular electrical stimulation from about 1.76 to 1.62 μm.

For sarcomere length measurement cells were placed in a Laminin coated, heated chamber (35–36°C) on an inverted microscope (ZeissAxiovert 100 TV, Jena, Germany, lens: Neofluar 40/0.75) and continuously perfused with the experimental solutions. Sarcomere shortening of isolated ventricular myocytes was recorded with a video imaging system (Myocam) and SarcLen software (IonOptix; Milton, MA, USA) as previously described (Figure 1A) (19). The regular striation pattern of the sarcomeres is analyzed by fast Fourier transformation. Sarcomere shortening shifts the power spectrum peak, which corresponds to the absolute sarcomere length (Figure 1C) (20). Shortenings were induced by bipolar external stimuli *via* two gold electrodes (0.4 ms, 40 V, SD9, Grass; Quincy, MA, USA). During the recordings, the isolated cardiomyocytes were continuously stimulated at a frequency of 4 Hz. After a “run-in period” of about 200 s to establish steady-state shortenings with continuous bathing with experimental Tyrode, the solution was switched to a drug-containing Tyrode for 3 min. Afterward, the solution was switched back to Tyrode solution again for 3 min to show wash-out of the drug (Figure 1B).

### Drugs

Stock solutions containing CBZ and LTG were dissolved in dimethyl sulfoxide (DMSO). LEV was prepared in distilled water. Stock solutions were stored at −20°C or prepared freshly and added to experimental Tyrode just before use. The final experimental concentrations used in our experiments were 100 μM CBZ, 100, 250, and 500 μM LTG, and 100 μM LEV. The concentration of DMSO did not exceed 1:1,000 (1:500 for LTG 500 μM). The Tyrode solution used before and after drug wash-in (control condition) contained DMSO in corresponding concentrations. Drugs and all other chemicals were purchased from Sigma-Aldrich (Taufkirchen, Germany).

### Data Analysis

The evaluation of the cardiomyocyte shortening was carried out using IonWizard 6 (IonOptix). Only rod shaped faultless cells with a uniform striation pattern were selected for recordings. Recordings were included in the final analysis when cardiomyocytes displayed a stable steady state at the point of data acquisition and when they survived the recurrent stimulation for the whole recording period. To exclude a toxic effect or gradual death of the recorded cardiomyocytes, we only included those recordings in which the drug effect was reversible. We assessed (i) the number of successful shortenings following individual electrical stimulations (electromechanical coupling) and (ii) the shortening properties (sarcomere length, shortening amplitude, maximal shortening velocity, maximal relengthening velocity, and the time to return to 90% of the baseline) upon repetitive electrical stimulation at 4 Hz before, during, and after application of the AEDs.

The shortening velocity (in micrometers per second) characterizes the maximal speed with which the cell shortens. The relengthening velocity (in micrometers per second) is the maximal speed in the return phase of the transient. The time to return to 90% of the baseline (in milliseconds) is a measure of cellular relaxation. To quantify the shortening properties, we have averaged the last 10 shortening signals before wash-in and wash-out, respectively. For statistical analysis, a repeated-measures ANOVA followed by Newman–Keuls corrected *post hoc* analysis was used when appropriate. ANOVA tests were performed using GraphPad Prism 6.0 (GraphPad Software Inc., San Diego, CA, USA). *p*-Value <0.05 was considered as significant (***p < 0.001, **p < 0.01, *p < 0.05). All data are given as mean ± SEM.

## Results

### External Ca^2+^ Reversibly Modulates Shortening Amplitudes of Isolated Cardiomyocytes

**Figure 2:**
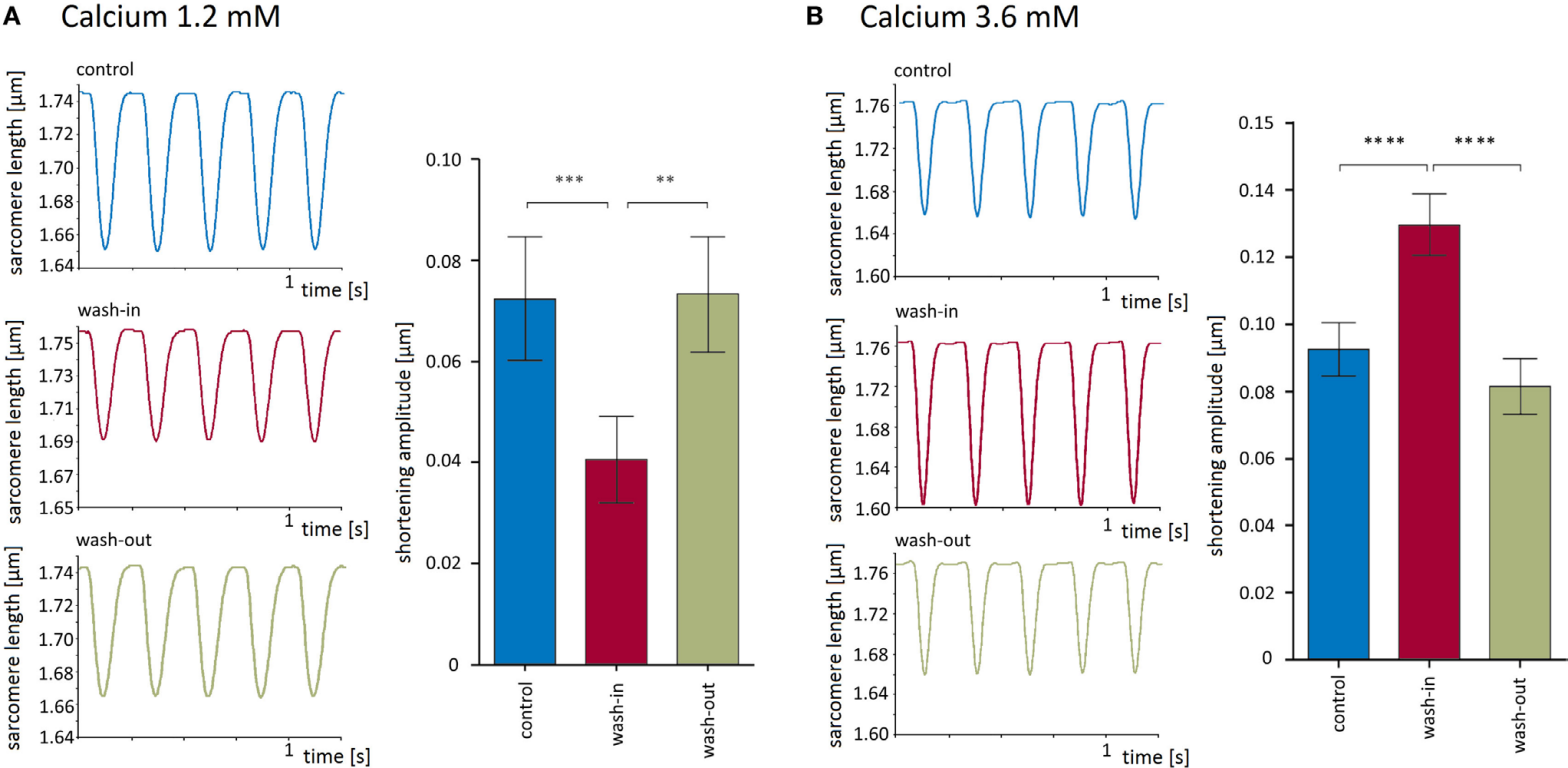
**Modulation of sarcomere shortening properties by external calcium**. **(A)** Lowering of external calcium to 1.2 mM reversibly impaired shortening amplitudes. Left panel shows recording traces before, during, and after switch to external solution containing 1.2 mM. The results of six recordings are summarized as bar charts in the right panel. **(B)** Increasing external calcium to 3.6 mM reversibly enhanced shortening amplitudes. Left panel shows recording traces before, during, and after switch to external solution containing 3.6 mM Ca^2+^. The results of 12 recordings are summarized as bar charts in the right panel. Both the Ca^2+^-dependent reduction and the respective increase in shortening are highly significant.

To explore the recording conditions and the validity of our experimental approach, we measured sarcomere shortening at three different external calcium concentrations, namely, 1.2, 1.8 (defined as control condition), and 3.6 mM. As expected, the switch from 1.8 to 1.2 mM external Ca^2+^ reversibly decreased the shortening amplitude of the isolated cardiomyocytes by 46 ± 5% (from 0.073 ± 0.012 μm under control condition to 0.041 ± 0. 009 μm, *p* < 0.001, Figure 2A). Conversely, the increase of the external Ca^2+^ concentration from 1.8 to 3.6 μM led to a reversible enhancement of the shortening amplitude by 44 ± 5% (from 0.093 ± 0.008 to 0.13 ± 0.01 μm, *p* < 0.0001, Figure 2B).

### AEDs Reversibly Impair Shortening Properties of Isolated Cardiomyocytes

**Figure 3:**
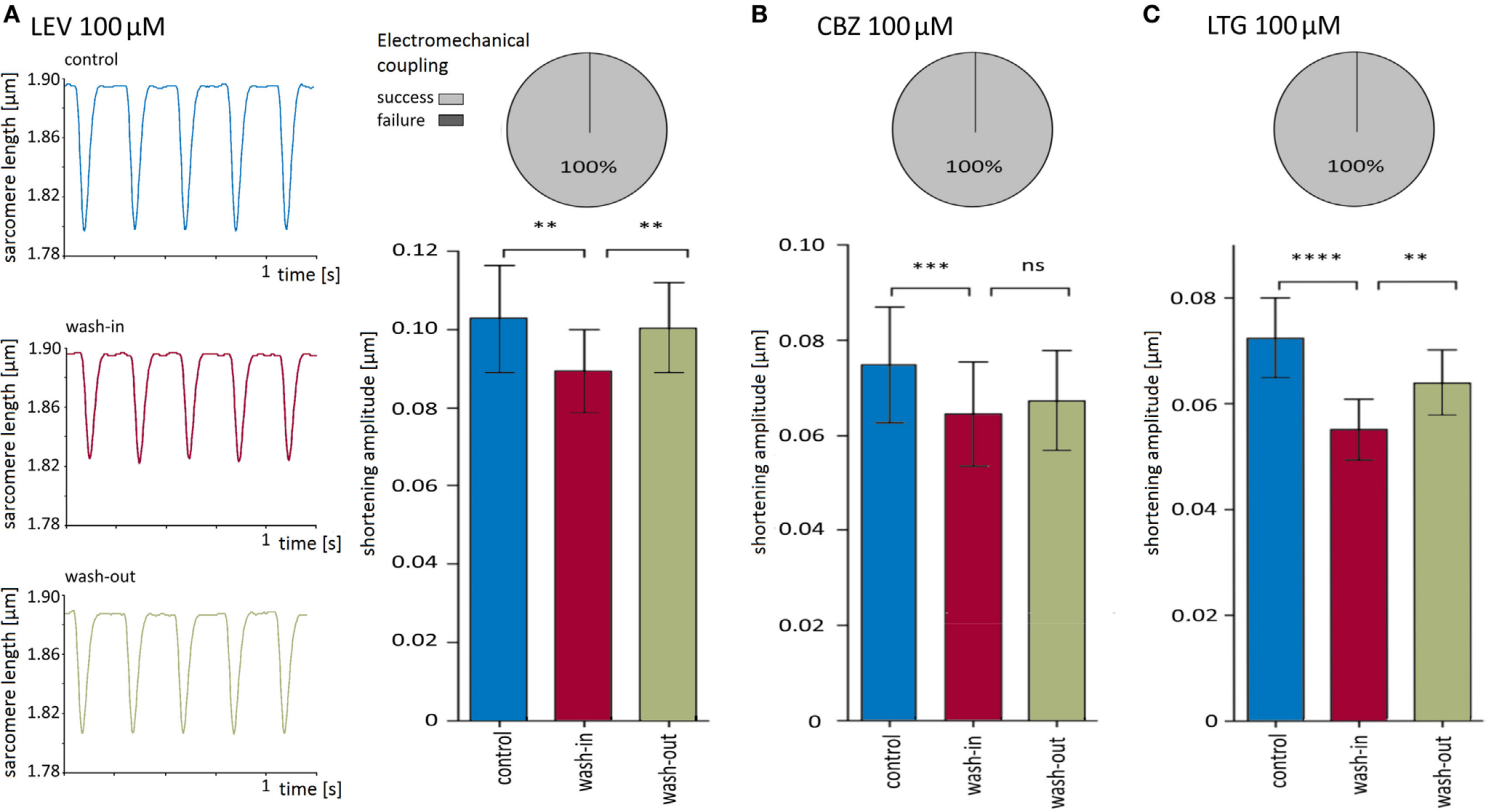
**Carbamazepine (CBZ), lamotrigine (LTG), and levetiracetam (LEV) reversibly reduced shortening amplitudes**. **(A)** Left panel shows original recording traces of one cell before, during and after wash-in of 100 μM LEV. The bar chart summarizes the significant and reversible reduction of shortening amplitudes of 11 cells, and the pie chart visualizes the efficacy of electromechanical coupling. **(B)** 100 μM CBZ (10 cells) also reduced significantly the shortening amplitude (bar chart) but did not influence excitation–shortening coupling (pie chart). **(C)** 100 μM LTG (11 cells) developed comparable effects as LEV and CBZ.

First, the effects on the shortening properties of cardiomyocytes were tested for each AED separately. All three drugs at a concentration of 100 μM did not affect electromechanical coupling (i.e., each electrical stimulation induced a shortening) but led to a reversible reduction in shortening amplitudes. Original recordings of sarcomere lengths and shortenings before, during, and after application of LEV are shown in Figure 3A. While the depressive effects of CBZ (10 cells) and LEV (11 cells) ranged between 11 and 15% (Figures 3A,B; Table 1), LTG (11 cells) led to a reduction of the shortening amplitude by about 24% (Figure 3C; Table 1). Furthermore, LTG also reduced the shortening velocity by 23 ± 4% and the relengthening velocity by 23 ± 5% (Table 1), whereas CBZ and LEV did not affect the shortening and relengthening velocities at the given concentration. The time of return to 90% of the baseline was not affected by any of the tested drugs.

**Table 1:** **Absolute effects of antiepileptic drugs on shortening properties**.

	Lamotrigine 100 μM (*n* = 11)	Carbamazepine 100 μM (*n* = 10)	Levetiracetam 100 μM (*n* = 11)	Combination of all 3 drugs (*n* = 11)
Shortening amplitude (μm)	−0.0174 ± 0.003	−0.0103 ± 0.0022	−0.0134 ± 0.0038	−0.0264 ± 0.0051
	***p* < 0.0001**	***p* < 0.001**	***p* < 0.01**	***p* < 0.001**
Shortening velocity (μm/s)	−0.778 ± 0.149	−0.3775 ± 0.2089	−0.6415 ± 0.277	−1.442 ± 0.269
	***p* < 0.001**	*p* > 0.05	*p* > 0.05	***p* < 0.0001**
Relengthening velocity (μm/s)	0.4733 ± 0.1363	0.1414 ± 0.1183	0.4669 ± 0.204	0.9769 ± 0.248
	***p* < 0.01**	*p* > 0.05	*p* > 0.05	***p* < 0.01**
Time to return to baseline 90% (ms)	−0.727 ± 4.433	2.80 ± 4.08	2.727 ± 3.14	6.455 ± 2.695
	*p* > 0.05	*p* > 0.05	*p* > 0.05	*p* > 0.05

*p-Values are compared to the control condition (before application of the drug)*.

*Significant p-values are highlighted in bold*.

**Figure 4:**
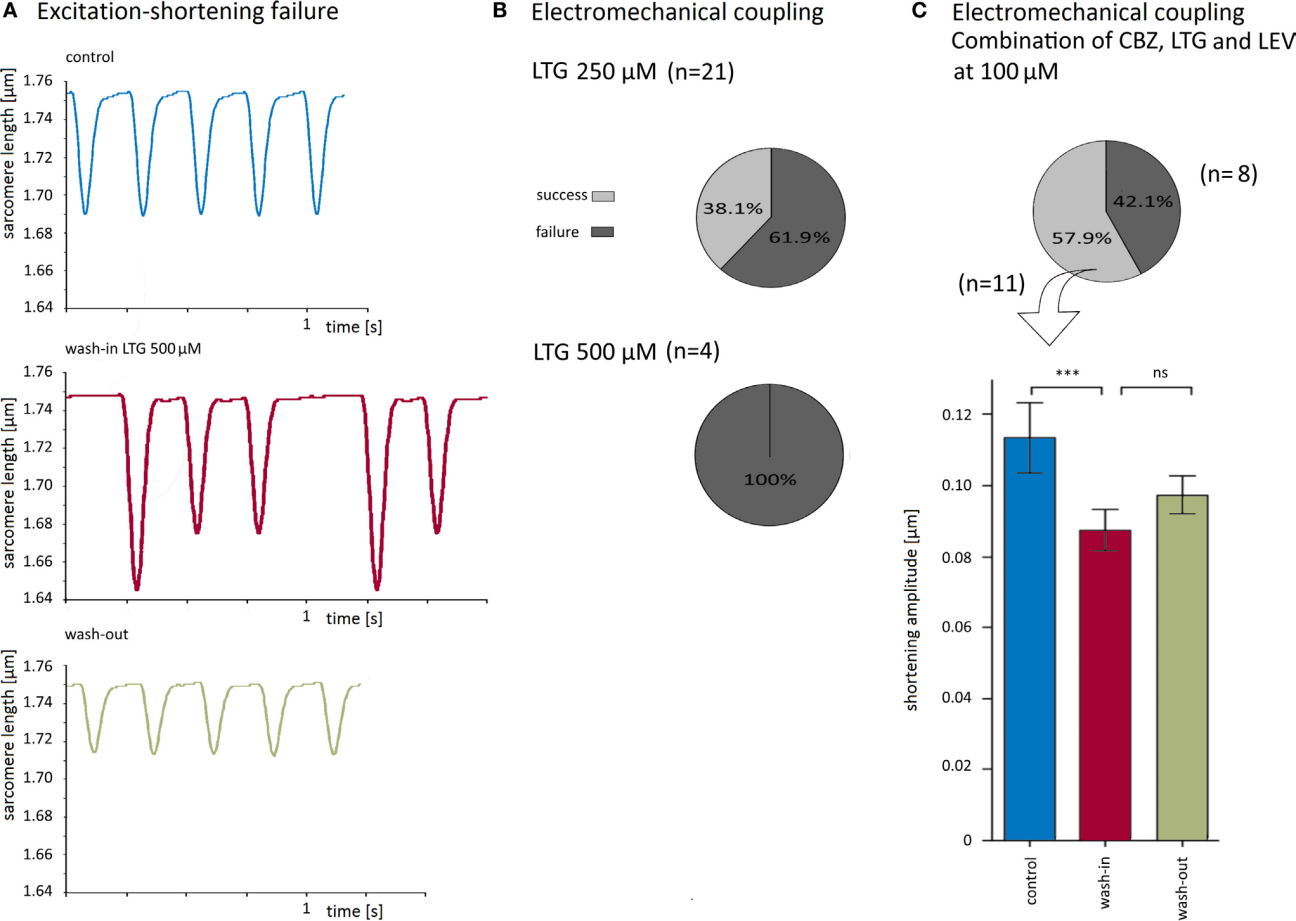
**Lamotrigine (LTG) at higher concentrations and simultaneous application of carbamazepine (CBZ), LTG, and levetiracetam (LEV) impaired electromechanical coupling**. **(A)** Recording traces before, during, and after wash-in of 500 μM LTG upon continuous electrical stimulation at 4 Hz. Note that shortening amplitudes were greater if precedent stimulations failed to evoke a shortening (middle panel). **(B)** At 250 and 500 μM LTG, the excitation–shortening coupling was blocked in 62 and 100% of the cells, respectively. **(C)** Simultaneous application of all 3 antiepileptic drugs at 100 μM blocked electromechanical coupling in 8 of 19 cells (pie chart). In cells without faulty excitation–shortening coupling, shortening amplitudes were reduced by about 21%.

As 100 μM LTG appeared to have the greatest impact on cardiomyocytes, we have also explored the effects of LTG at higher concentrations (250 and 500 μM). Interestingly, during application of LTG at 250 (21 cells) and 500 μM (4 cells), a variable proportion of the cardiomyocytes failed to shorten in response to every single electrical stimulation (for original traces see Figure 4A). The number of the failed shortenings increased with increasing time of application. At 250 and 500 μM LTG, the electromechanical coupling was irregular in 62 and 100% of the cells, respectively (Figure 4B). The shortening amplitudes after a failed excitation in 500 μM LTG were higher than those under control conditions, because murine cardiomyocytes develop an increased post-rest shortening after a stimulation pause compared to the steady state.

### Effect of Simultaneously Applied AEDs on Electromechanical Coupling

So far, we have applied the AEDs separately. In clinical practice, however, people with epilepsy often take more than one AED to achieve better seizure control. Therefore, CBZ, LTG, and LEV were simultaneously applied at a concentration of 100 μM each, i.e., at the same concentration as compared to the abovementioned experiments when each AED was applied alone. The combination of all three drugs led to electromechanical coupling failure in eight cells (42% of all recordings, Figure 4C). In the 11 cells with undisturbed coupling, effects on the shortening properties of the simultaneous drug application could be properly determined. The shortening amplitude was inhibited by 21 ± 4% (Figure 4C), the shortening and relengthening velocities were reduced by 23 ± 3 and 18 ± 5%, respectively (Table 1).

**Figure 5:**
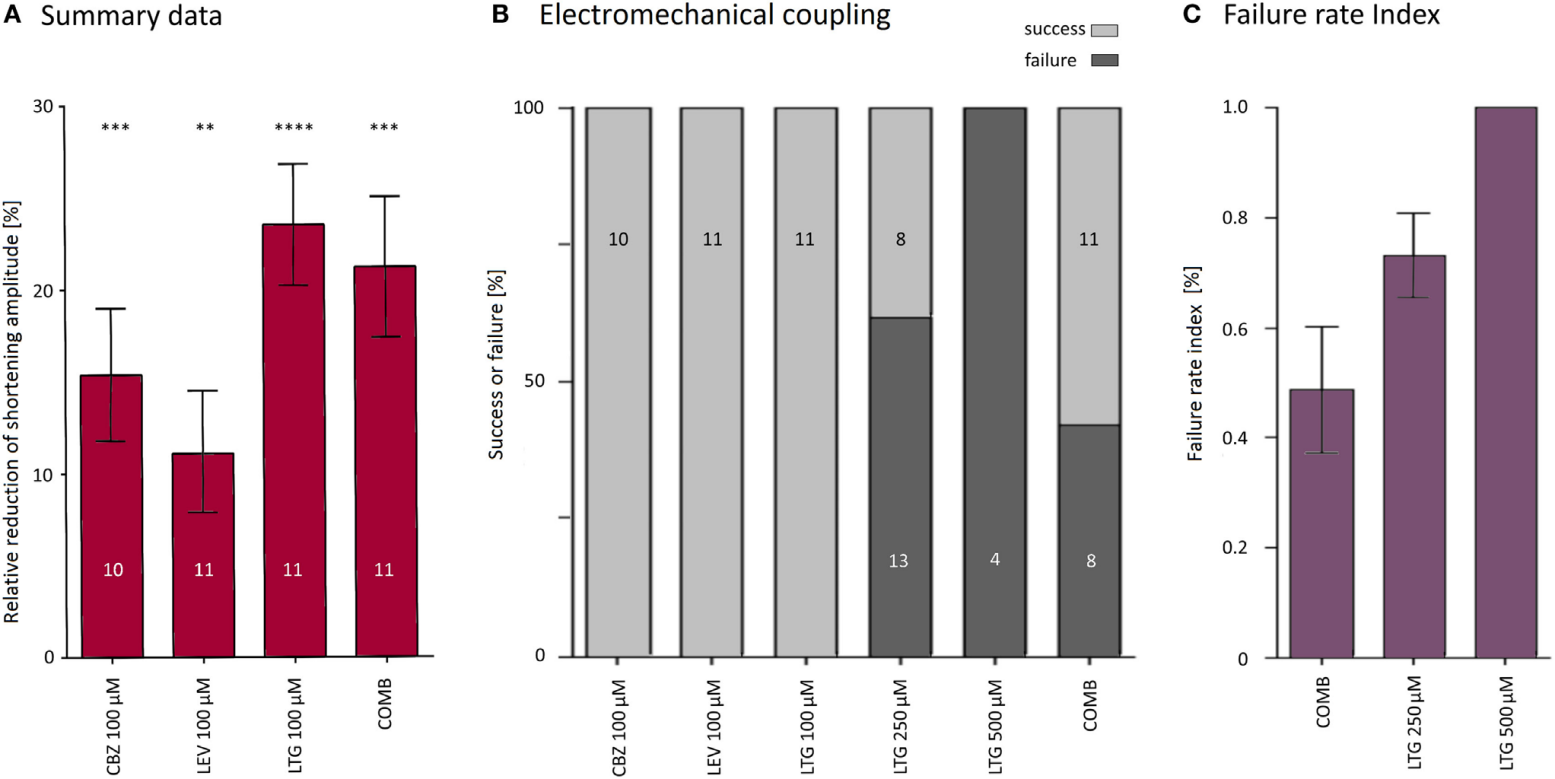
**Summary of antiepileptic drug (AED) effects on shortening properties**. **(A)** AEDs alone or in combination reduced shortening amplitudes by about 11–24%. **(B)** Only lamotrigine (LTG) at higher concentrations and simultaneous application of all three AEDs blocked excitation–shortening coupling. **(C)** The proportion between the number of failed stimulations (i.e., stimulation without subsequent shortening) and the total number of stimulations was defined as failure rate index. About 50% of the stimulations did not evoke shortenings in the presence of all three AEDs (COMB).

To further characterize the blocking effects on the electromechanical coupling, we have quantified the number of failed stimulations (i.e., no shortening) with respect to the total number of electrical stimulations for those cells that showed a disturbed electromechanical coupling. The failure rate amounted to 49 ± 11% upon simultaneous application of CBZ, LEV, and LTG (i.e., every second stimulation failed to evoke a shortening) and was even higher during application of very high LTG concentrations (Figure 5C).

## Discussion

### Depressed Cardiac Shortening: Mechanisms of Action and Possible Clinical Implications

All three AEDs alone reversibly reduced the shortening amplitudes of isolated cardiomyocytes (Figure 5A). Our study was not designed to investigate the mechanisms that are involved in the modulation of shortening properties of isolated cardiomyocytes. In the case of CBZ and LTG, however, the well-described inhibitory effects on VGSC and VGCC most likely underlie the observed depressive action of CBZ and LTG (12, 13). CBZ and LTG at 100 μM reduced the shortening amplitudes by about 15 and 24%, respectively. It is difficult to translate the extent and importance of these *in vitro* findings into clinical practice for at least two reasons: first, we have applied relatively high concentrations of CBZ and LTG as compared to the typical therapeutic serum levels in epilepsy patients (LTG 12–58 μM, CBZ 17–51 μM) (21). Second, the inhibitory effects of CBZ and LTG on VCSC are use dependent, i.e., the inhibition gets stronger with increasing stimulation frequencies (22, 23). In our experiments, the cardiomyocytes were stimulated at 4 Hz only, as the recordings were stable for at least 10–15 min at this particular frequency. This stimulation frequency equals a heart rate of 240 beats per minute, which is low as compared to the habitual resting heart rate between 500 and 600 beats per minute in mice, but still much higher than the resting heart rate in adult humans. Thus, the effect size of CBZ or LTG on cardiac contraction at typical therapeutic concentrations is likely to be lower in epilepsy patients. In the instance of a CBZ or LTG overdose, however, the inhibitory effects on contraction properties as well as on electromechanical coupling (as shown at 250 and 500 μM LTG) appear to be of clinical importance, as illustrated by previous case reports (24, 25).

Levetiracetam at 100 μM reversibly reduced the shortening amplitude by about 11%. This depressive LEV effect was rather unexpected but can be explained by its inhibitory action on voltage-gated L-type calcium channels (26, 27). Although the concentration of LEV was well within the typical serum levels of epilepsy patients (70–270 μM) (21), the cardiodepressive effect is small and unlikely to be clinically significant, given the fact that rapid intravenous infusion of LEV had no apparent cardiovascular effects in healthy controls and epilepsy patients (28, 29).

### Faulty Electromechanical Coupling: Mechanisms of Action and Possible Clinical Implications

Electromechanical coupling was reversibly blocked only at very high concentrations of LTG or when all three AEDs at 100 μM were simultaneously applied (Figures 5B,C). In our experimental constellation, this phenomenon may be due to an inhibition of cellular excitation, of the excitation–shortening coupling, the shortening itself, or a combination of these elements, probably *via* binding to VGSC and VGCC.

In our point of view, the effect of the simultaneous application of the AEDS is particularly important in the clinical context. About one-third of the epilepsy patients are difficult to treat, i.e., they commonly need more than one AED to achieve better or full seizure control (30). Polypharmacy, in turn, was suspected to increase the risk of SUDEP (31). Subsequent studies have finally revealed that polypharmacy is not a risk factor itself, but rather a surrogate marker of severe epilepsy and insufficient seizure control (32) and that efficacious adjunctive AED treatment reduces the SUDEP risk (33). It appears, however, plausible that simultaneous administration of AEDs that bind to the same cellular targets increases the risk of target-related side effects. Therefore, it is not surprising that cardiac side effects such as atrioventricular block, sinus node dysfunction, or arrhythmogenic ST–T abnormalities were reported in the context of simultaneous intake of AED that inhibits VGSC (34–36). We think that cardiac side effects can indeed, if long lasting and severe enough, facilitate sudden cardiac death as a rare cause of SUDEP in some epilepsy patients. This view is also supported by the finding that AED intake is an independent risk factor for sudden cardiac death (10, 11). In this context, it is of note that CBZ and LTG were discussed as potential risk factors for SUDEP in previous studies (17). However, these assumptions could not be replicated in subsequent meta-analyses or when studies were adjusted for the frequency of generalized tonic–clonic seizures (17, 32, 37). To date, no single AED has unequivocally been proven to increase the SUDEP risk.

### Study Limitations and Translational Value of Our Findings for SUDEP in Humans

People with epilepsy often display acquired or genetic alterations of voltage-gated ionic channels that are expressed in both heart and brain (6, 38). In the present study, however, we have tested the effects of three AEDs on the shortening properties of isolated cardiomyocytes of wild-type mice only, but not on cardiomyocytes of mice with genetic or acquired epilepsy. In this context, it is of note that significant disturbances of electrical properties were found in cardiomyocytes of rodents with acquired or genetic epilepsies. For instance, the expression of NaV1.1 was increased in ventricular cardiomyocytes of a rat model of acquired epilepsy (kainic acid-induced status epilepticus model), leading to a prolonged duration of cardiac action potential and most likely to a prolongation of the QT interval (39). In a mouse model of Dravet syndrome, a severe form of childhood epilepsy with high mortality which is commonly caused by heterozygous mutations in NaV1.1, ventricular myocytes surprisingly exhibited an increase in voltage-dependent sodium currents, probably due to an enhanced expression of NaV1.5 (40). These alterations were associated with an increased excitability, a prolongation of action potential duration, and an increase in triggered activity of cardiomyocytes in mice carrying the Dravet mutation. These two examples nicely illustrate possible modifications of cardiac properties at the level of gene expression in animal models of epilepsy but also demonstrate the difficulties in predicting potential consequences of such alterations for AED effects. A measurable effect on modulation of voltage-gated sodium currents by AED in cardiomyocytes of humans or animals with epilepsy, however, seems likely, given the differential sensitivities of splice variants and types of VGSC to AEDs (23, 41, 42). Thus, future studies are required to compare the effects of AEDs at different concentrations (including other AEDs than CBZ, LEV, and LTG) on cardiomyocytes of wild-type mice to those of mice with an epileptic phenotype.

Given these considerations, the translational value of our study for SUDEP in humans is rather limited. In view of the large spectrum of epilepsy-related cardiorespiratory alterations, SUDEP is likely to be a heterogeneous phenomenon and to have multiple etiologies (4, 5). Therefore, it appears plausible that in some cases, SUDEP is caused by fatal cardiac dysfunction due to myocardial failure, bradyarrhythmias, or ventricular tachyarrhythmias. Here, we have shown that CBZ, LEV, and LTG reversibly reduced the shortening of isolated wild-type cardiomyocytes and that LTG at higher concentrations or simultaneous application of all three AEDs blocked electromechanical coupling. Our findings may at least partially explain some of the cardiac disturbances, which were reported in epilepsy patients treated with AEDs in mono- or polytherapy. For instance, myocardial failure was reported in the context of CBZ intoxication (24, 43, 44), a complete atrioventricular block upon an overdose of LTG (25) and occurrence of sinus node dysfunction, atrial flutter/fibrillation, atrioventricular block, or ventricular tachycardia in association with the combined use of sodium channel-blocking AEDs (34, 35, 45, 46).

In summary, our results suggest that rationale AED therapy may be important with respect to cardiac side effects and potential facilitation of serious cardiac dysfunction especially when AEDs are used in combination or at very high doses.

## Ethics Statement

The animal handling and care conformed to the German federal laws and to NIH Guide for the Care and Use of Laboratory Animals published by the US National Institutes of Health (NIH Publication No. 85-23, revised 1996).

## Author Contributions

JH has performed the experiments and the primary data analysis and contributed to the writing of the manuscript. CE has contributed to interpretation of the data and revised the manuscript critically for important intellectual content. RM has supervised the experiments and contributed to the study design, data analysis, and writing of the manuscript. RS has contributed to the study design, data analysis, and writing of the manuscript.

## Conflict of Interest Statement

CE has served as a paid consultant for UCB Pharma, Desitin, and Pfizer. He is an employee of the Life and Brain Institute Bonn. RS has received fees as speaker or consultant from Bial, Cyberonics, Desitin, EISAI, Novartis, and UCB Pharma. JH and RM have no potential conflicts of interest.
